# The Plaque Analysis Classifies the Coronary Artery Disease‐Reporting and Data System (CAD‐RADS) Stenosis and Plaque Burden Categories: Association of the Plaque Features, Fat Attenuation Index, Coronary Computed Tomography Fractional Flow Reserve, and the Combination of Stenosis and Calcification

**DOI:** 10.1002/clc.24305

**Published:** 2024-06-17

**Authors:** Wenxi Chen, Jiyan Nie, Mingyu Zhang, Zhi Zhu, Yuanyong Zhou, Qingde Wu, Xuxia He

**Affiliations:** ^1^ Graduate School Guangzhou University of Chinese Medicine Guangzhou China; ^2^ Department of Radiology Shunde Hospital of Guangzhou University of Chinese Medicine Shunde China

**Keywords:** coronary atherosclerosis, fractional flow reserve, plaque, plaque burden

## Abstract

**Background:**

The coronary artery disease‐reporting and data system (CAD‐RADS) 2.0 is used to standardize the reporting of coronary computed tomography angiography (CCTA) results. Artificial intelligence software can quantify the plaque composition, fat attenuation index, and fractional flow reserve.

**Objective:**

To analyze plaque features of varying severity in patients with a combination of CAD‐RADS stenosis and plaque burden categorization and establish a random forest classification model.

**Methods:**

The data of 100 patients treated between April 2021 and February 2022 were retrospectively collected. The most severe plaque observed in each patient was the target lesion. Patients were categorized into three groups according to CAD‐RADS: CAD‐RADS 1−2 + P0−2, CAD‐RADS 3−4B + P0−2, and CAD‐RADS 3−4B + P3−4. Differences and correlations between variables were assessed between groups. AUC, accuracy, precision, recall, and F1 score were used to evaluate the diagnostic performance.

**Results:**

A total of 100 patients and 178 arteries were included. The differences of computed tomography fractional flow reserve (CT‐FFR) (*H* = 23.921, *p* < 0.001), the volume of lipid component (*H* = 12.996, *p* = 0.002), the volume of fibro‐lipid component (*H* = 8.692, *p* = 0.013), the proportion of lipid component volume (*H* = 22.038, *p* < 0.001), the proportion of fibro‐lipid component volume (*H* = 11.731, *p* = 0.003), the proportion of calcification component volume (*H* = 11.049, *p* = 0.004), and plaque type (*χ* ^2^ = 18.110, *p* = 0.001) was statistically significant.

**Conclusion:**

CT‐FFR, volume and proportion of lipid and fibro‐lipid components of plaques, the proportion of calcified components, and plaque type were valuable for CAD‐RADS stenosis + plaque burden classification, especially CT‐FFR, volume, and proportion of lipid and fibro‐lipid components. The model built using the random forest was better than the clinical model (AUC: 0.874 vs. 0.647).

## Graphical abstract

Computed tomography fractional flow reserve, volume and proportion of lipid and fibro‐lipid components of plaques, proportion of calcified components, and plaque type were valuable for coronary artery disease‐reporting and data system stenosis + plaque burden classification. Notably, the above model built using random forest is better than clinical model (area under curve: 0.874 vs. 0.647).

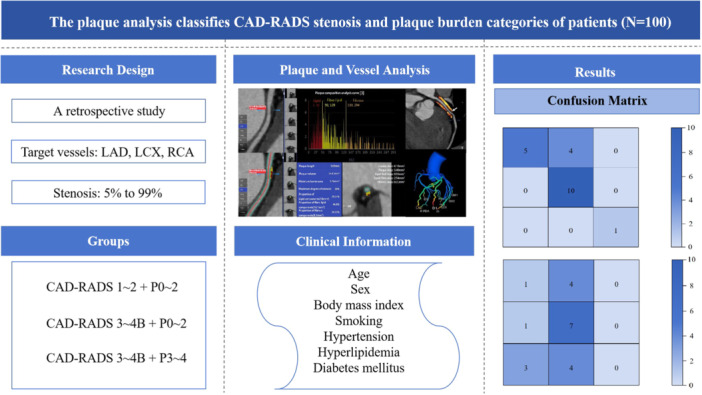

**Abbreviations**
AI: artificial intelligence
AUC: area under the curve
CAD‐RADS: coronary artery disease‐reporting and data system
CFD: computational fluid dynamics
CT‐FFR: coronary CT fractional flow reserve
FAI: fat attenuation index
ICC: intergroup correlation coefficient

## Advances in Knowledge

The CAD‐RADS categorization and plaque burden were considered as a whole to evaluate the patients, and the level of inflammation, fractional flow reserve, and plaque features of the target lesion were analyzed simultaneously and comprehensively based on the level of stenosis and degree of calcification.

## 1 Introduction

In the field of coronary imaging, invasive fractional flow reserve (FFR) is the gold standard for the assessment of functional myocardial ischemia [1]. Currently, the coronary CT angiography‐derived fractional flow reserve (CT‐FFR) is calculated using the computational fluid dynamics (CFD) method without additional imaging or vasodilator administration to provide blood pressure and flow throughout the coronary tree [2]. Not satisfied with only the CFD method, advanced deep learning combined with CFD algorithms has been used to optimize the performance of CT‐FFR [3] and has already been confirmed through studies to be as reliable as invasive FFR [4, 5, 6]. In recent years, researchers have also found that the fat attenuation index (FAI), a novel imaging marker, reflects the inflammation of pericoronary fat [7, 8, 9, 10], and the inflammatory factors of pericoronary fat in turn interact informatively with plaque. The coronary artery disease‐reporting and data system (CAD‐RADS) standardizes the categorization of patients with coronary artery disease based on the severity of stenosis and is widely used in clinical practice for diagnosis and risk prediction [4, 11]. Researchers have employed a convolutional neural network algorithm to classify CAD‐RADS, which significantly reduces the time required for CCTA diagnosis [12]. However, the relationship between myocardial ischemia, level of vascular inflammation, and plaque features with CAD‐RADS categorization in patients with plaques in their vessels remains to be explored. Interestingly, the interaction of the vessel wall and plaque is complicated, as reflected in their microbiological and macroscopic mechanical characteristics, and adverse events in patients with coronary atherosclerosis are not due to stenosis [13]. Therefore, in this study, we combined CAD‐RADS stenosis categorization with plaque burden categorization and analyzed the plaques of the most severe lesions in terms of composition, length, and volume, as well as CT‐FFR and FAI values in patients with different categorizations to discover the relationship between the individual patient and the local target lesion and establish a random forest classification model.

## 2 Methods and Materials

### 2.1 Study Population

This retrospective study included patients who underwent CCTA examinations at Shunde Hospital of Guangzhou University of Chinese Medicine from April 2021 to February 2022. The study was conducted in accordance with the principles of the Declaration of Helsinki. The inclusion criteria were: (1) coronary atherosclerosis diagnosed by CCTA. (2) The target arteries were the left anterior descending (LAD), left circumflex (LCX), and right coronary arteries (RCA) [14]. (3) The stenosis range was 5%−99%. (4) Complete imaging workstation data. A total of 108 patients were excluded based on the following criteria: (1) Poor coronary image quality, artifacts, or noise (*n* = 31). (2) Errors in AI recognition, including unrecognition, incomplete recognition, and misrecognition of artifacts as plaques (*n* = 13). (3) Coronary artery slenderness [15] (diameter less than 1.8 mm) (*n* = 9). (4) History of coronary stent implantation or cardiac surgery (*n* = 25). (5) Patients with severe cardiovascular disease (severe heart failure or severe arrhythmia, etc.) (*n* = 16). (6) Incomplete clinical information (*n* = 14). Ultimately, 100 patients were enrolled in this study.

### 2.2 Scanning Protocol

All patients were scanned with a 3rd‐generation dual‐source CT Force (Healthineers, Siemens, Germany) and received sublingual nitroglycerin (0.5 mg/tablet; Beijing Yimin Pharmaceutical Co., Ltd.) 3−5 min before the examination. The specific scanning protocol was consistent with the routine CCTA clinical practice. Iopromide (60 mL) (370 mg I/mL, Bayer, Germany) was injected via an elbow venous high‐pressure syringe, and 40 mL intravenous saline was used for washout at a flow rate of 5 mL/s. A prospective electrocardiographic trigger sequence was used, and the scan was automatically triggered when the CT threshold at the level of the aortic arch below the tracheal bifurcation reached 120 HU. The scan was performed from 1 cm below the carina of the trachea to 2 cm below the diaphragm. Other scan parameters were as follows: tube voltage of 100 kV, automatic tube current, collimator width of 96 × 0.6 mm, rack speed of 0.25 s/revolution, reconstruction layer thickness of 0.80 mm, and layer spacing of 0.5 mm.

### 2.3 Image Quality Assessment

The images were uploaded to the quantitative coronary CT image stenosis analysis software (CoronaryDoc, Shukun Network Technology Company, Beijing, China). The software first evaluated the CCTA image quality, which was divided into five grades using AI: grade 5 image quality was excellent; grade 4 was good; grade 3 was moderate; grade 2 was very poor; and grade 1 was not diagnostic. To ensure the accuracy of the AI in identifying plaque components, images with a quality below grade 3 were not included.

### 2.4 Plaque Analysis

CoronaryDoc software automatically identifies segmented coronary arteries according to proprietary algorithms and generates maximum intensity projections, multiplanar reconstruction, and reconstruction using body‐drawing techniques. After the AI workstation recognizes the vessel and lumen boundaries, we manually correct them if necessary. Subsequently, the target lesion plaque length, volume, plaque type (calcified, non‐calcified, and partially calcified plaques), plaque composition (volume and proportion of lipid, fibrous, fibrous‐lipid, and calcified components), minimum lumen area, and degree of luminal stenosis were determined. When multiple plaques were present, we selected non‐calcified plaques as the most severe stenoses for analysis.

Plaque type was defined by the presence or absence of a calcified component in the plaque; lipid, fibro‐lipid, fibrous, and calcified components were classified as follows: <50 HU for the lipid component; 50−130 HU for the fibro‐lipid component; 130−350 HU for the fibrous component; and ≥350 HU for the calcified component. Noncalcified plaque volume = lipid component + fiber component + fibro‐lipid component volume; partially calcified plaque volume = lipid component + fiber component + fibro‐lipid component + calcified component volume. The plaque components were delineated using enhanced CT images.

According to Oikonomou et al. [9], FAI was calculated for perivascular fat in the proximal segment of 4 cm; CT‐FFR based on three‐dimensional CFD combined with deep learning was presented in the form of a colored coronary tree, with different colors denoting different CT‐FFR values, and the measurement site was located 2 cm posterior to the stenosis of the target lesion.

### 2.5 Identification of High‐Risk Plaques

Two readers performed conventional diagnosis on the images under double‐blinded conditions to determine the presence of high‐risk factors in the plaque (spotty calcification, low‐density plaque, “napkin ring” sign, measurement of remodeling index, and determination of positive remodeling) and whether it was a high‐risk plaque. High‐risk factors and high‐risk plaques were defined as follows: (1) spotty calcification was defined as calcification with a CT value higher than 130 HU in non‐calcified plaque, and the length of calcification less than 3 mm in any plane; (2) low‐density plaque referred to regions with CT value lower than 30 HU in non‐calcified plaque; (3) the “napkin‐ring” sign indicated a plaque with a low‐density core in the adjacent lumen and high‐density surrounding; (4) the ratio of the maximal diameter of the plaque to its average diameter of the proximal and distal arteries is remodeling index ratio (manual measurement), with a ratio of 1.1 or higher indicating positive remodeling. The simultaneous presence of more than two of the above risk factors was considered a high‐risk plaque [16].

### 2.6 Statistical Analysis

SPSS 25.0 IBM and Python 3.8.1 were used for statistical analysis. Normality was assessed using the Shapiro–Wilk test for measurement information. Quantitative information with normal distribution was expressed as $\bar{x}\pm s$, non‐normally distributed was expressed as M (Q1, Q3), and qualitative information was expressed as number of cases and percentage. The diagnostic consistency between the two readers was evaluated using the intergroup correlation coefficient (ICC). Comparison of quantitative information between groups was performed using the ANOVA test or Kruskal−Wallis *H* test, and the *χ* ^2 ^test or Fisher's exact test was used for comparisons of qualitative data between groups. Kendall's correlation coefficient was used to analyze the correlation between CAD‐RADS, plaque burden, and myocardial ischemia. Random forest was applied to build the model, and the AUC, accuracy, precision, recall, and F1 score were used to evaluate the efficiency of each model. All statistical tests were two‐sided, and statistical significance was set at *p* < 0.05.

## 3 Results

### 3.1 General Information

A total of 100 patients with 178 arteries were included, including a total of 87 (48.88%) LAD, 35 (19.66%) LCX, and 56 (31.46%) RCA. A single artery was involved in 46 (46%) patients, two arteries in 30 (30%), and three arteries in 24 (24%). A total of 6 (6%) had superficial myocardial bridges in the LAD, but no deep myocardial bridges were observed. All image qualities were of grades 3−5. The basic patient characteristics are summarized in Table 1.

**Table 1:** Basic characteristics of patients (*n* = 100).

Characteristics		
Age, years		62.60 ± 11.28
Sex, male, *n* (%)		53 (53)
Body mass index, kg/m^2^		23.85 (22.40−26.18)
Hypertension, *n* (%)		65 (65)
Diabetes mellitus, *n* (%)		24 (24)
Hyperlipidemia, *n* (%)		21 (21)
Smoking, *n* (%)		20 (20)
CAD‐RADS		
	1	8
	2	29
	3	37
	4A	23
	4B	3
Plaque burden		
	P0	27
	P1	42
	P2	16
	P3	10
	P4	5
Myocardial ischemia		
	I−	64
	I+	9
	I+/−	27

Abbreviation: CAD‐RADS, coronary artery disease reporting and data system.

### 3.2 Correlation among CAD‐RADS, Plaque Burden, and Myocardial Ischemia

Comparing the correlations among the CAD‐RADS category, plaque burden, and myocardial ischemia, which were analyzed using Kendall's correlation coefficient, as all three variables represent graded information, we found statistically significant correlations between each pairwise combination of the three, and a moderate positive correlation was found between myocardial ischemia and CAD‐RADS grading based on stenosis (*r* = 0.535, *p* < 0.001) (Table 2). Of these, the CT‐FFR was consistent with the FFR (*r* = 0.772, *p* < 0.001).

**Table 2:** Kendall's correlation coefficient analysis.

	*r*	*p* Value
CAD‐RADS & myocardial ischemia	0.535	<0.001
CAD‐RADS & plaque burden	0.273	<0.001
Plaque burden & myocardial ischemia	0.290	<0.001

Abbreviation: CAD‐RADS, coronary artery disease reporting and data system.

### 3.3 Comparison of FAI, CT‐FFR, and Plaque Features Between Groups

There was a high concordance between the results of the two readers in determining high‐risk factors and high‐risk plaque features (ICC = 0.830, 95% CI: 0.758−0.883, *p* < 0.001). In our study, the FFR was measured in 42 patients. We compared the CT‐FFR of this group of patients with the FFR and found a remarkable agreement between the two (*r* = 0.772, *p* < 0.001). We categorized CAD‐RADS into grades 1−2 and 3−4B and plaque burden into grades P0−2 and 3−4, and then combined them two‐by‐two as a grouping of patients in our study. Since there were no patients in the group of CAD‐RADS 1−2 + P3−4, we categorized them into three groups. Compared between groups on FAI, CT‐FFR, and plaque features, we found the differences of CT‐FFR (*H* = 23.921, *p* < 0.001), the volume of lipid component (*H* = 12.996, *p* = 0.002), the volume of fibro‐lipid component (*H* = 8.692, *p* = 0.013), the proportion of lipid component volume (*H* = 22.038, *p* < 0.001), the proportion of fibro‐lipid component volume (*H* = 11.731, *p* = 0.003), the proportion of calcification component volume (*H* = 11.049, *p* = 0.004), and plaque type(*χ* ^2^ = 18.110, *p* < 0.001) was statistically significant (Table 3).

**Table 3:** Comparison between groups on FAI, CT‐FFR, and plaque features.

	CAD‐RADS 1−2 + P0−2	CAD‐RADS 3−4B + P0−2	CAD‐RADS 3−4B + P3−4	Statistic value	*p*
	(*n* = 37)	(*n* = 48)	(*n* = 15)		
FAI	−77.95 ± 7.31	−79.04 ± 9.55	−80.01 ± 8.92	0.357^a^	0.701
CT‐FFR	0.95 (0.88−0.98)	0.83 ± 0.12	0.73 ± 0.13	23.921^b^	<0.001
Minimum lumen area (mm^2^)	1.75 (0.59−3.15)	2.94 (0.79−3.81)	3.52 (1.20−5.06)	4.675^b^	0.097
Plaque features					
Length (mm)	6.44 (3.58−10.52)	8.21 (5.37−13.51)	8.79 (5.41−16.06)	3.506^b^	0.173
Volume (mm^3^)	10.36 (2.63−27.76)	19.76 (8.93−39.51)	17.93 (7.86−41.96)	5.103^b^	0.078
Lipid (mm^3^)	0.47 (0−2.77)	1.70 (0.99−7.65)	0.11 (0−4.91)	12.996^b^	0.002
Fibro‐lipid (mm^3^)	2.60 (0−10.10)	6.03 (2.77−12.68)	1.09 (0−6.40)	8.692^b^	0.013
Fibrous (mm^3^)	3.62 (0−11.28)	5.96 (1.84−16.56)	1.46 (0−16.20)	2.944^b^	0.229
Calcification (mm^3^)	1.73 (0.26−6.27)	1.30 (0−5.62)	9.09 (0−27.99)	5.473^b^	0.065
Lipid (%)	3.37 (0−8.91)	15.10 (5.50−27.28)	1.07 (0−12.16)	22.038^b^	<0.001
Fibro‐lipid (%)	25.22 ± 21.59	35.32 (25.27−48.25)	5.60 (0−37.89)	11.731^b^	0.003
Fibrous (%)	31.61 (0−47.43)	30.45 ± 17.74	26.22 (0−43.02)	1.420^b^	0.492
Calcification (%)	18.04 (5.20−100)	6.85 (0−19.68)	40.50 (0−100)	11.049^b^	0.004
Plaque type				18.110^c^	0.001
Calcified plaque, *n* (%)	10 (27.03)	3 (6.25)	6 (40)		
Non‐calcified plaque, *n* (%)	22 (59.46)	39 (81.25)	4 (26.67)		
Partially calcified plaque, *n* (%)	5 (13.51)	6 (12.50)	5 (33.33)		
High‐risk plaque	3 (8.11)	5 (10.42）	3 (20)	1.692^c^	0.509
Spotty calcification, *n* (%)	3 (8.11)	1 (2.08)	2 (13.33)	3.321^c^	0.155
Low‐density plaque, *n* (%)	0	5 (10.42)	1 (6.67)	4.165^c^	0.122
Napkin‐ring sign, *n* (%)	0	2 (4.17)	0	1.562^c^	0.641
Positive remodeling index	1.25 ± 0.16	1.24 (1.15−1.40)	1.29 ± 0.31	0.630^b^	0.730
Positive remodeling, *n* (%)	31 (83.78)	43 (89.58)	12 (80.00)	1.111^d^	0.574

Abbreviations: CT‐FFR, coronary CT flow reserve fraction; FAI, fat attenuation index.

^a^ The *F* value of the ANOVA test.

^b^ The *H* value of the Kruskal−Wallis *H* test.

^c^ The *χ* ^2^ value of Fisher's exact test.

^d^ *χ* ^2^ value of the chi‐square test.

### 3.4 Built Models of Random Forests to Distinguish Groups

**Figure 1:**
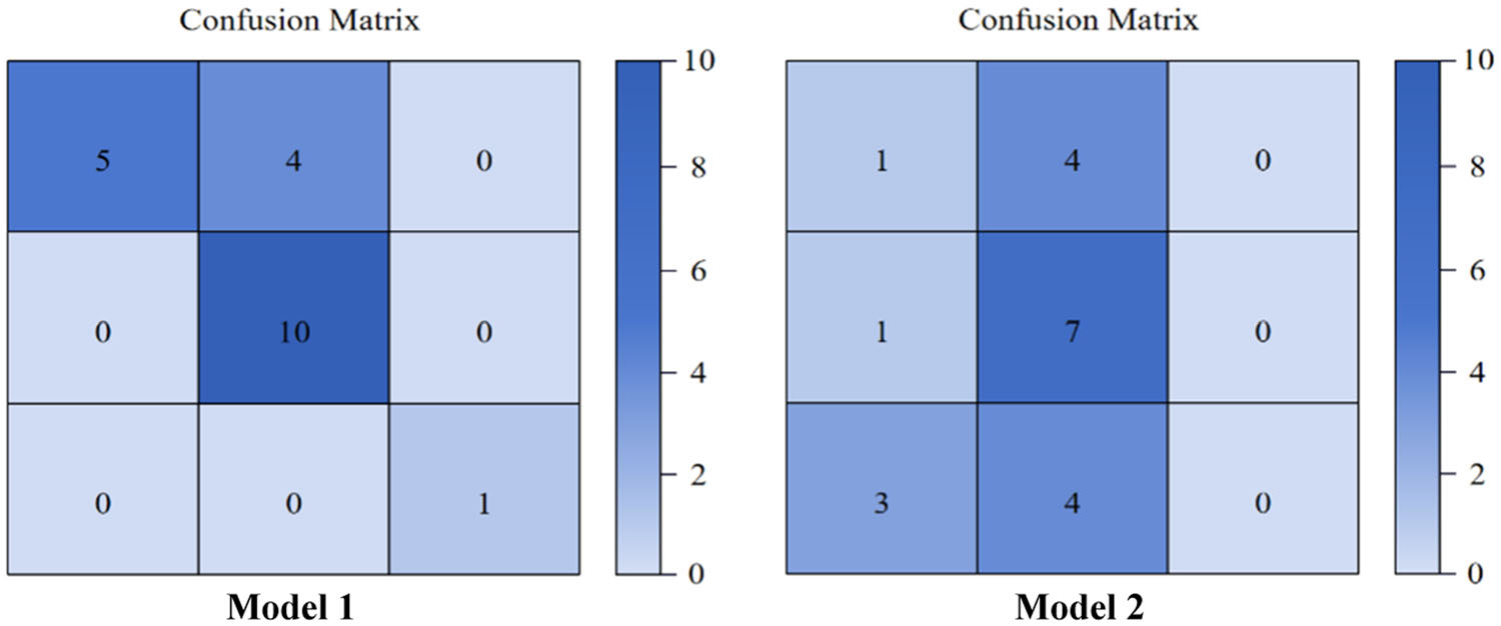
Confusion matrix of random forest in the test set.

Using the random forest algorithm, we employed the statistically significant variables listed in Table 3 for modeling, achieving an AUC of 0.874 (model 1). Additionally, a clinical model that incorporated factors such as age, sex, smoking history, hyperlipidemia, hypertension, and diabetes mellitus was developed. This clinical model (model 2) was constructed using the random forest algorithm, yielding an AUC of 0.647. The efficiencies of both models are presented in Table 4, and the confusion matrix is illustrated in Figure 1.

**Table 4:** Predictive efficacy of random forest.

	AUC	Accuracy	Precision	Recall	F1 score
Model 1	0.874	0.800	0.905	0.852	0.849
Model 2	0.647	0.400	0.222	0.358	0.270

*Note:* Model 1, adjustment for CT‐FFR, the volume of lipid component, the volume of fibro‐lipid component, the proportion of lipid component volume, the proportion of fibro‐lipid component volume, the proportion of calcification component volume, and plaque type. Model 2, adjustment for age, gender, smoking history, hyperlipidemia, hypertension, and diabetes mellitus.

Abbreviation: AUC, area under curve.

## 4 Discussion

This retrospective cohort study integrated patient CAD‐RADS categorization with plaque burden grading to assess plaques at different severity levels. The plaques examined in this study represented the most severe lesions in the LAD, LCX, and RCA arteries of the patients. The CAD‐RADS, as a standardized reporting tool, can provide a standard framework for diagnostic imaging physicians. Although CAD‐RADS version 2.0, released in 2022, focuses on the importance of modifiers, such as plaque burden and myocardial ischemia, each remains a separate unit. In our study, for the first time, we considered both the CAD‐RADS categorization and plaque burden to comprehensively evaluate patients. Simultaneously, we analyzed the level of inflammation, flow reserve capacity, and plaque characteristics of the target lesion, considering both the levels of stenosis and calcification, thereby encompassing both CAD‐RADS stenosis and plaque burden categorization.

In this study, we discovered that CAD‐RADS grades 3−4 B exhibited a larger volume and proportion of lipid or fibro‐lipid components than CAD‐RADS grades 1−2, specifically among individuals with a lower plaque burden (P0−2). These findings partially align with those reported by Kinoshita et al. [17], who employed Optical Coherence Tomography to identify vulnerable plaques. They concluded that higher CAD‐RADS scores indicated higher inflammation levels and plaque vulnerability. Conversely, the plaque components associated with plaque vulnerability include thin fibrous caps, large lipid necrotic cores encompassing more than 10% of the plaque area, and macrophage infiltration. Large lipid plaque ruptures [18] and uneven fibrous cap thickness with surface irregularities [19] may indicate plaque instability. From a biological stress perspective, alterations in blood pressure at the stenosis site provoked notable deformation of the lipid and fiber interface within the plaque [20]. This deformation subsequently alters blood flow patterns, ultimately resulting in myocardial ischemia. Furthermore, the findings of our study demonstrated that an increase in plaque burden was associated with a decrease in the mean CT‐FFR value, particularly in cases of high CAD‐RADS stenosis degree (3−4B) when we compared the groups of “CAD‐RADS 3−4B + P0−2” and “CAD‐RADS 3−4B + P3−4.” A greater degree of calcification tended to correlate with a lower CT‐FFR value [20].

However, despite being a subjective qualitative indicator, high‐risk plaques were not significant in our study. We primarily used it to differentiate between the severity of stenosis and the extent of calcification rather than as a determinant of overall risk. CAD‐RADS 2.0, which emphasizes that the presence of high‐risk plaques is more closely associated with non‐obstructive lesions or the occurrence of specific ischemia. Moreover, high‐risk plaques play a crucial role in patients with acute coronary syndromes [21].

Based on the previously mentioned CT‐FFR, plaque type, and meaningful plaque composition features, we developed a CAD‐RADS+ plaque burden grading model using a random forest algorithm. Moreover, CT‐FFR, the volume share of lipid composition, and fibro‐lipid composition contributed the most to the CAD‐RADS+ plaque burden grading. Clinical factors were also modeled routinely, with an AUC of weaker efficacy than those of the models based on CT‐FFR and plaque features. Dai et al. [22] also analyzed the clinical factors of patients and discovered that individuals with a CAD‐RADS categorization of 3−5 exhibited a higher prevalence of hyperlipidemia, diabetes mellitus, and hypertension than those classified as 0−2, which is consistent with our findings. Subsequently, they trained a model using 19 features to predict the CAD‐RADS score and demonstrated that plasma fibrinogen, age, and history of diabetes, with higher feature weights, contributed significantly to the model's performance, achieving an AUC of 0.832.

In addition, patients with a high grade of CAD‐RADS + plaque burden had a greater target plaque length and volume, although there was no statistically significant difference in our study. In some studies [23, 24], ischemic vessels had larger plaque volumes and longer plaque lengths. There was a negative correlation between these characteristics and both quantitative stenosis and FFR, which is consistent with the findings of the present study. Correlation analyses revealed a moderate association between the CAD‐RADS grade and myocardial ischemia, indicating that a higher degree of stenosis in the vessel and a greater number of vessels involved increased the likelihood of myocardial ischemia.

In our study, a CT‐FFR constructed using deep learning combined with CFD modeling was applied. The three‐dimensional rigid model of CFD alone partially neglected the effect of artery wall motion on fluid dynamics. In addition, the study has the following limitations: first, the sample included only 100 patients from a single center, requiring a larger sample size for constructing a random forest model; second, because not all patients required invasive coronary angiography, the consistency test of invasive FFR and CT‐FFR was performed only on certain patients; third, because of variations in the clinical relevance of total coronary artery occlusion depending on the clinical background, patients with CAD‐RADS 5 grade were not included; fourth, although there was a significant difference in FAI between patients with grade 4A or less and grade 4B or more [17], grade 4B was not subdivided in this study because only three patients had this grade. The study's strengths are: (1) high image quality, less susceptible to artifacts; (2) high diagnostic specificity and sensitivity of AI, offering reliable identification of plaques and stenosis; and (3) all data stem from real plaques, ensuring that the statistical results are real and reliable.

## 5 Conclusion

In conclusion, CT‐FFR, the volume and proportion of lipid and fibro‐lipid components of plaques, the proportion of calcified components, and plaque type were valuable for CAD‐RADS + plaque burden classification, especially CT‐FFR, volume, and proportion of lipid and fibro‐lipid components. The model built using the random forest performed better than the clinical model (0.874 vs. 0.647).

## Author Contributions

**Wenxi Chen:** research design, data collection, data organization, statistical analysis, and paper writing. **Jiyan Nie:** research design, data collection, implementation of the study, and statistical analysis. **Mingyu Zhang and Zhi Zhu:** data collection, data organization, statistical analysis. **Yuanyong Zhou:** implementation of the study, paper review, and research supervision. **Qingde Wu:** research design, paper review, and research supervision. **Xuxia He:** implementation of the study, paper review, and research supervision.

## Ethics Statement

Ethical review and approval were waived for this study due to the retrospective study.

## Consent

Patient consent was waived due to a retrospective study (KY‐2024040).

## Conflicts of Interest

The authors declare no conflicts of interest.

## Data Availability

Data are not available due to ethical restrictions.
